# Characterization of the Complete Mitochondrial Genome of *Tachypleus tridentatus* and Mitochondrial Phylogenetic Analysis of Extant Limulidae

**DOI:** 10.3390/ani16182823

**Published:** 2026-09-08

**Authors:** Lei Xu, Xingzhi Zhang, Yafang Li, Quehui Tang, Jiajia Ning, Lianggen Wang, Shuangshuang Liu, Delian Huang, Dapeng Wang, Pinyuan Wei, Feiyan Du, Jinxia Peng

**Affiliations:** 1 South China Sea Fisheries Research Institute, Chinese Academy of Fishery Sciences, Guangzhou 510300, China; xulei@scsfri.ac.cn (L.X.); xiaofang-1113@163.com (Y.L.); tangquehui@scsfri.ac.cn (Q.T.); njpiao1981@163.com (J.N.); lgwang@scsfri.ac.cn (L.W.); liuss1029@163.com (S.L.);; 2 Guangxi Academy of Fishery Sciences, Nanning 530021, China; xingzhi.zh@foxmail.com (X.Z.);

**Keywords:** Limulidae, mitogenome, molecular phylogeny, *Tachypleus tridentatus*, horseshoe crab

## Abstract

The tri-spine horseshoe crab, *Tachypleus tridentatus*, is an evolutionarily ancient marine chelicerate of considerable ecological and conservation importance in the Indo-West Pacific. Although several mitochondrial genomes have been reported, comprehensive comparative mitogenomic analyses encompassing all extant limulid genera remain limited. In this study, we sequenced and characterized the complete mitochondrial genome of an F1 *Tachypleus tridentatus* individual derived from wild-caught broodstock from the Beibu Gulf, northern South China Sea, and subsequently maintained at an aquaculture facility, using high-throughput sequencing, and performed an integrated comparative mitogenomic analysis across the three extant genera of Limulidae. The circular mitochondrial genome was 15,006 bp in length and contained the typical set of 37 mitochondrial genes, including 13 protein-coding genes (PCGs), 22 transfer RNA genes, and two ribosomal RNA genes. The newly sequenced *Tachypleus tridentatus* mitogenome showed highly conserved genome organization and nucleotide composition relative to previously published conspecific mitogenomes, confirming established mitochondrial characteristics of the species. The principal contribution of this study is the addition of a complete Beibu Gulf mitogenome and its integration with 12 additional complete mitochondrial genomes representing all extant limulid species, together with four mitochondrial markers, K2P genetic-distance analyses, and independent BI and ML phylogenetic reconstruction. The concordance among these mitochondrial datasets provides an integrated assessment of genetic divergence and mitochondrial phylogenetic relationships within Limulidae and expands the geographic representation of complete mitochondrial genomic resources for *Tachypleus tridentatus*.

## Simple Summary

Horseshoe crabs are ancient marine animals that have lived on Earth for hundreds of millions of years. They play an important role in coastal ecosystems and are also valuable for medical testing. However, their populations have declined sharply in recent decades due to habitat loss and human activities. In this study, we sequenced and analyzed the full set of mitochondrial genetic material from a tri-spine horseshoe crab from the Beibu Gulf in southern China. We compared it with genetic data from other horseshoe crab species around the world to better understand their evolutionary relationships. Our results confirm that the genetic structure of horseshoe crabs is highly conserved, and we provide a new genetic reference for this regional population. This work will help future studies of horseshoe crab diversity and support conservation efforts for these endangered ancient creatures.

## 1. Introduction

Horseshoe crabs are among the most ancient extant marine arthropods and represent the only surviving lineage of the class Xiphosura. Fossil evidence indicates that the evolutionary history of horseshoe crabs can be traced back approximately 450 million years to the Ordovician period, making them one of the oldest animal groups still existing on Earth [1,2,3]. Throughout geological history, horseshoe crabs have survived multiple global extinction events, including the end-Permian and end-Cretaceous mass extinctions, while retaining a remarkably conserved body morphology characterized by a horseshoe-shaped prosoma, opisthosoma, and elongated telson. This exceptional morphological stability over hundreds of millions of years has earned horseshoe crabs the designation of “living fossils” [4,5]. Their long evolutionary persistence and relatively unchanged body plan provide a unique opportunity to investigate evolutionary stasis, adaptation, and lineage survival over geological timescales. As members of Chelicerata, horseshoe crabs occupy a pivotal position in arthropod evolution. Traditionally, Xiphosura has been regarded as the sister group to Arachnida based on morphological and developmental characteristics [6]. However, recent phylogenomic studies employing large-scale molecular datasets have challenged this traditional view and suggested alternative hypotheses in which horseshoe crabs may be nested within Arachnida or form a more complex evolutionary relationship with other chelicerate lineages [7,8]. These conflicting phylogenetic hypotheses have generated considerable interest in resolving the evolutionary placement of horseshoe crabs and understanding the origin and diversification of chelicerates [9]. Consequently, horseshoe crabs have become important model organisms in studies of evolutionary biology, comparative genomics, developmental genetics, and phylogenetics. The accumulation of genomic resources from horseshoe crabs is therefore essential for clarifying their evolutionary history and for improving our understanding of arthropod evolution as a whole [10,11].

Currently, only four extant horseshoe crab species are recognized worldwide, namely *Tachypleus tridentatus*, *Tachypleus gigas*, *Carcinoscorpius rotundicauda*, and *Limulus polyphemus*, all belonging to the family Limulidae. Among these species, three occur in Asia, whereas *L. polyphemus* is distributed along the Atlantic coast of North America. The present-day diversity of horseshoe crabs is remarkably low compared with the extensive fossil diversity documented throughout the Paleozoic and Mesozoic eras, suggesting that extant species represent the surviving remnants of a once much more diverse evolutionary lineage. The family Limulidae therefore constitutes an evolutionarily significant group for investigating patterns of diversification, extinction, and biogeographic history within Xiphosura [12,13,14]. Among extant limulid species, *T. tridentatus* is the largest and one of the most widely distributed horseshoe crabs in the Indo-West Pacific region. The species occurs along the coastal waters of China, Japan, Taiwan, and Vietnam, inhabiting estuarine environments, shallow coastal waters, and intertidal mudflats [15,16]. As a benthic ecosystem engineer, *T. tridentatus* contributes to nutrient cycling, sediment bioturbation, and energy transfer within coastal food webs [17]. In addition to its ecological significance, the species possesses considerable economic and biomedical value because horseshoe crab amebocyte lysate has been extensively used for bacterial endotoxin detection in pharmaceutical and medical industries. However, rapid coastal urbanization, habitat degradation, land reclamation, pollution, and overexploitation have resulted in substantial population declines throughout much of its distribution range [18,19,20]. Consequently, *T. tridentatus* is currently listed as an endangered species and has become one of the primary targets of horseshoe crab conservation efforts in Asia. Understanding the evolutionary history and genetic characteristics of this species is therefore of both scientific and conservation importance [21,22].

Mitochondrial genomes have become one of the most informative molecular resources for investigating evolutionary relationships, molecular evolution, and population genetics in animals. The typical metazoan mitochondrial genome is a circular DNA molecule ranging from 14 to 20 kb in length and generally contains 37 genes, including 13 protein-coding genes (PCGs), 22 transfer RNA genes (tRNAs), and two ribosomal RNA genes (rRNAs), together with a major non-coding control region involved in the regulation of replication and transcription [23,24]. Compared with nuclear genomes, mitochondrial genomes possess several advantageous characteristics, including maternal inheritance, compact genome organization, limited recombination, relatively rapid evolutionary rates, and high copy numbers within cells. These features make mitochondrial DNA particularly useful for species identification, phylogenetic reconstruction, phylogeographic inference, population genetic analyses, and conservation studies [25,26,27,28,29]. With the rapid development of next-generation sequencing technologies, complete mitochondrial genomes have been increasingly employed to investigate evolutionary patterns across diverse taxonomic groups [24,30,31,32,33]. Mitochondrial genomes are particularly informative for investigating the evolutionary history of horseshoe crabs because their compact organization and relatively conserved gene content provide a tractable framework for examining evolutionary stability in this ancient lineage [23,24]. At the same time, mitochondrial sequences retain sufficient variation to resolve lineage divergence and maternal evolutionary history among extant limulid taxa [25,26,34,35]. These characteristics are especially relevant to horseshoe crabs, which exhibit remarkable morphological conservatism over geological timescales and are widely regarded as “living fossils”. Comparative mitogenomic analyses can therefore complement morphological evidence by revealing molecular patterns of lineage differentiation that may not be apparent from conserved external morphology [34,35]. In addition, *T. tridentatus* has experienced substantial population declines associated with habitat loss, degradation, and overexploitation. Mitochondrial genomic resources can therefore provide useful molecular references for species identification, lineage characterization, and subsequent population-level conservation genetic studies. Thus, complete mitogenomes are valuable not only for resolving phylogenetic relationships within Limulidae but also for establishing a genomic baseline for understanding the evolutionary history and conservation of these evolutionarily distinctive marine arthropods.

Despite the ecological and evolutionary significance of horseshoe crabs, important gaps remain in the available mitogenomic resources for *T. tridentatus* and other limulids. Although complete mitochondrial genomes have been reported for several horseshoe crab species, previous studies have largely focused on genome sequencing, annotation, and phylogenetic reconstruction, whereas comprehensive comparisons of mitochondrial genome organization, nucleotide composition, codon usage, and evolutionary dynamics across Limulidae remain limited [36,37,38]. Moreover, prior population genetic studies of *T. tridentatus* along the Chinese coast and Taiwan Strait have mainly relied on short mitochondrial markers, such as COI and other variable mitochondrial loci, and have revealed low but detectable geographic genetic differentiation among some populations [39,40]. However, complete mitogenomic data remain geographically uneven, and, to our knowledge, no complete mitochondrial genome from *T. tridentatus* associated with the Beibu Gulf region had been available prior to this study. This geographic gap is particularly relevant because the Beibu Gulf represents an important habitat region for *T. tridentatus*, yet molecular resources for assessing regional genetic variation remain limited [41]. Generating a complete mitogenome from the Beibu Gulf region therefore provides a geographically informative reference for comparative mitogenomics and establishes an additional molecular resource for future investigations of geographic population differentiation and conservation genetics. In the present study, we sequenced, assembled, and characterized the complete mitochondrial genome of *T. tridentatus*. Detailed analyses were conducted to investigate genome organization, nucleotide composition, codon usage patterns, and the structural characteristics of transfer RNA genes and non-coding regions. To place the newly generated genome in a broader evolutionary context, comparative analyses were performed using available mitogenomic data from representatives of all extant genera of Limulidae, followed by mitochondrial genetic-distance and phylogenetic analyses. These analyses provide new insights into mitochondrial genome evolution and lineage relationships within Limulidae while expanding the geographic representation of mitogenomic resources available for *T. tridentatus*.

## 2. Materials and Methods

### 2.1. Sample Collection and DNA Extraction

Multiple F1 larvae of *T. tridentatus* were obtained from the aquaculture facility of Guangxi Academy of Fishery Sciences, Guangxi, China. These larvae were F1 offspring produced from wild-caught broodstock maintained for artificial breeding, rather than individuals derived from a long-term captive-bred lineage. No wild individuals were collected specifically for the present study. The use of F1 offspring derived from wild-caught broodstock avoided additional collection pressure on wild populations while providing access to genetic material derived from wild individuals. Because the specimens represented the first filial generation, they were less likely to have experienced cumulative genetic changes associated with prolonged captive propagation, such as genetic drift and artificial selection over multiple generations. Nevertheless, the number and genetic composition of the broodstock may influence the representation of mitochondrial haplotypes in the F1 offspring. Species identification was confirmed based on morphological characteristics following established taxonomic descriptions. One representative individual was selected for mitochondrial genome sequencing. Body muscle tissue samples were excised, immediately preserved in 95% ethanol, and subsequently stored at −80 °C until DNA extraction. All procedures involving the use of Tachypleus tridentatus specimens were reviewed and approved by the Animal Ethics Committee of the Guangxi Academy of Fishery Sciences (Approval No. 2026-GAFS-27). The specimens used in this study were F1 offspring produced from wild-caught broodstock and maintained at an established aquaculture facility. No additional wild individuals were collected specifically for this study. The experimental procedures were limited to tissue sampling, genomic DNA extraction, PCR amplification, and mitochondrial genome sequencing, without experimental intervention, treatment, or behavioral manipulation of live animals. All procedures were conducted in accordance with the institutional requirements for the ethical use and handling of animal-derived biological materials and applicable national regulations. The study also complied with applicable requirements of the Convention on Biological Diversity (CBD) and the Nagoya Protocol regarding the use of biological resources. Total genomic DNA was extracted from body muscle tissue using the TIANamp Marine Animals DNA Kit (TIANGEN, Beijing, China) following the manufacturer’s instructions. The specimen and the extracted DNA have been stored at the South China Sea Fisheries Research Institute, Chinese Academy of Fishery Sciences, registered under the voucher number SCS2026-T1-210. DNA quality and concentration were assessed using 1% agarose gel electrophoresis and a NanoDrop 2000 spectrophotometer (Thermo Scientific, Waltham, MA, USA). The extracted genomic DNA had a concentration of 20 ng/μL and an A260/A280 ratio of 1.80. DNA integrity was evaluated by agarose gel electrophoresis (Thermo Scientific, Waltham, MA, USA), and the DNA was considered suitable for subsequent library preparation and mitochondrial genome sequencing.

### 2.2. Mitochondrial Genome Sequencing, Assembly, and Annotation

Genomic DNA was randomly fragmented to approximately 200–400 bp by sonication. Fragmented DNA was subjected to end repair, A-tailing, and adapter ligation sequentially. The double-stranded libraries were subsequently denatured into single-stranded DNA and circularized. Residual linear DNA molecules were removed by exonuclease digestion. The resulting single-stranded circular DNA libraries were amplified by rolling circle amplification (RCA) to generate DNA nanoballs (DNBs). The DNB libraries were subsequently sequenced on the DNBSEQ-T7 platform (MGI Tech Co., Ltd., Shenzhen, China) using a paired-end 150 bp (PE150) sequencing strategy. Raw sequencing reads were filtered to remove adapter sequences, low-quality reads, and potential contaminants to obtain clean reads. The mitochondrial genome was assembled using GetOrganelle v1.7.7 [42], which is specifically designed for organelle genome assembly from next-generation sequencing data. The assembled mitochondrial genome was further polished using Pilon v1.24 to correct potential sequencing errors and improve sequence accuracy [43]. The complete mitochondrial genome sequence was annotated using the MITOS2 web server (http://mitos2.bioinf.uni-leipzig.de/index.py) with default parameters [44]. The annotation results were manually inspected and corrected using the mitochondrial genome of *Tachypleus tridentatus* (NC_012574.1) as a reference to ensure the accuracy of gene boundaries and annotation results. For genes with ambiguous annotations, additional verification was performed against the NCBI non-redundant (NR) protein database. The final mitochondrial genome annotation was manually curated and used for subsequent comparative and phylogenetic analyses.

To assess the reliability of the mitochondrial genome assembly, clean reads were mapped back to the assembled mitochondrial genome using BWA v0.7.17-r1188 [45]. The resulting alignments were processed using Samtools v1.5 to calculate the sequencing depth and genome-wide coverage [46]. The average sequencing depth and the proportion of mitochondrial genome positions covered by sequencing reads were calculated to quantitatively evaluate assembly completeness and sequencing support. Transfer RNA (tRNA) genes were predicted using tRNAscan-SE 1.21 via the online platform with default parameters and the “Mito/Chloroplast” search mode [47]. The mitochondrial genome annotation was performed based on the assembled and manually curated sequence data. Protein-coding genes and ribosomal RNA genes were identified by comparison with published mitochondrial genomes of related horseshoe crab species. A circular mitochondrial genome map was generated using the CGView Server to visualize gene organization and genomic features, including gene arrangement and GC content distribution [48].

### 2.3. Nucleotide Composition and Genetic Distance Analysis

The nucleotide composition of the mitochondrial genome was analyzed using MEGA software version 6.0 [49]. The frequencies of adenine (A), thymine (T), guanine (G), and cytosine (C) were calculated for the complete mitochondrial genome, protein-coding genes (PCGs), ribosomal RNA genes, transfer RNA genes, and the control region, respectively. Strand asymmetry was evaluated by calculating AT-skew and GC-skew using the following formulas: AT-skew = (A − T)/(A + T) and GC-skew = (G − C)/(G + C) [50]. The relative synonymous codon usage (RSCU) values of the 13 protein-coding genes were calculated using MEGA software version 6.0 to assess codon usage patterns in the mitochondrial genome. Codon usage analyses were based on the invertebrate mitochondrial genetic code. To assess genetic divergence among the three extant genera of Limulidae (*Tachypleus*, *Carcinoscorpius*, and *Limulus*), pairwise evolutionary distances were calculated using the Kimura two-parameter (K2P) model implemented in MEGA version 6.0. Both intra- and intergeneric genetic distances were estimated and summarized as mean and median values. The analysis was based on mitochondrial protein-coding genes (*COX1* and *Cytb*) and ribosomal RNA genes (12S rRNA and 16S rRNA). Gaps and missing positions were treated using pairwise deletion, so that only homologous nucleotide sites available for each sequence pair were included in the distance calculations. Pairwise distance matrices were generated separately for each gene and for the combined dataset to evaluate genetic divergence patterns across different mitochondrial markers. All sequence alignments were performed using MUSCLE implemented in MEGA prior to distance estimation.

To assess evolutionary constraints acting on mitochondrial protein-coding genes, the 13 PCGs from all available horseshoe crab mitochondrial genomes were extracted and aligned individually using MAFFT v7 [51]. The numbers of synonymous substitutions (Ks), nonsynonymous substitutions (Ka), and Ka/Ks ratios were calculated using DnaSP v6.0 [52]. The Ka/Ks ratio was used as an indicator of selective pressure, where Ka/Ks < 1 indicates purifying selection, Ka/Ks = 1 suggests neutral evolution, and Ka/Ks > 1 indicates positive selection. Comparative analyses of Ka/Ks values among mitochondrial genes were conducted to identify genes exhibiting different evolutionary rates and selective constraints.

### 2.4. Phylogenetic Analysis

To investigate the phylogenetic relationships within Limulidae, 13 complete mitochondrial genome sequences representing the four extant horseshoe crab species (*T. tridentatus*, *T. gigas*, *C. rotundicauda*, and *L. polyphemus*) were retrieved from the NCBI GenBank database. These sequences included representatives from different geographic populations and independent GenBank records. The mitochondrial genome of *Ixodes hexagonus* (GenBank accession number AF081828) was selected as the outgroup (Table S1). The nucleotide sequences of the 13 mitochondrial protein-coding genes (PCGs) were extracted from each mitogenome, and stop codons were excluded prior to analysis. Individual PCGs were aligned separately using MAFFT v7 under codon-based alignment settings, with the nucleotide sequences aligned according to their codon structure rather than translated into amino-acid sequences. Ambiguously aligned regions were identified and removed using Gblocks under default settings. The resulting codon-aware nucleotide alignments were concatenated into a combined nucleotide dataset using PhyloSuite v1.2.3 [53]. ModelFinder was used to determine the optimal partitioning scheme and nucleotide substitution models based on the Bayesian Information Criterion (BIC). For the BI analysis, the optimal partitioning scheme comprised three partitions: Partition 1 included *ATP6*, *ATP8*, *COX1*, *COX2*, *COX3*, *ND2*, and *ND3* and was assigned the GTR+F+G4 model; Partition 2 included *Cytb* and *ND6* and was assigned the GTR+F+I+G4 model; and Partition 3 included *ND1*, *ND4L*, *ND4*, and *ND5* and was assigned the GTR+F+I+G4 model. Bayesian inference was conducted using MrBayes v3.2.6 [54] with two parallel runs for 701,100 generations, and the initial 25% of sampled trees were discarded as burn-in. For the ML analysis, ModelFinder identified three optimal partitions under BIC: Partition 1 (*ATP6*, *ATP8*, *COX1*, *COX2*, *COX3*, *ND2*, and *ND3*) was assigned TIM2+F+G4; Partition 2 (*Cytb* and *ND6*) was assigned TIM2+F+I+G4; and Partition 3 (*ND1*, *ND4L*, *ND4*, and *ND5*) was assigned TIM+F+I+G4. Maximum-likelihood phylogenies were inferred using IQ-TREE v1.6.8 under the selected partitioning scheme, with 50,000 ultrafast bootstrap replicates and the Shimodaira–Hasegawa-like approximate likelihood-ratio test (SH-aLRT) used to evaluate nodal support [55]. Phylogenetic trees generated from the ML and BI analyses were visualized and edited using FigTree v1.4.4.

## 3. Results

### 3.1. Mitochondrial Genome Structure of Tachypleus Tridentatus

High-throughput sequencing generated a total of 5.97 Gb of raw sequencing data for the *T. tridentatus* specimen, with 95.16% of bases having a Phred quality score ≥ Q30. After quality filtering, the clean reads were used for mitochondrial genome assembly. The mitochondrial genome was assembled into a complete circular molecule using GetOrganelle and subsequently polished using Pilon. To assess assembly accuracy, clean reads were mapped back to the assembled mitochondrial genome using BWA, and sequencing depth was calculated using Samtools. The assembled mitochondrial genome showed an average sequencing depth of 374×, with 100% of the mitochondrial genome positions covered by sequencing reads, and no obvious coverage gaps were detected (Supplementary Figure S1). These results provided quantitative support for the completeness and reliability of the assembled mitochondrial genome. The complete mitochondrial genome of *T. tridentatus* was 15,006 bp in length (GenBank accession number: PZ744148), with an overall nucleotide composition of A 37.90%, T 36.13%, G 8.90%, and C 17.07%. The mitochondrial genome exhibited a strong AT bias, with an overall AT content of 74.03%. The AT-skew and GC-skew values were 0.0239 and −0.3146, respectively, indicating a slight excess of adenine over thymine and a pronounced bias toward cytosine over guanine.

**Figure 1:**
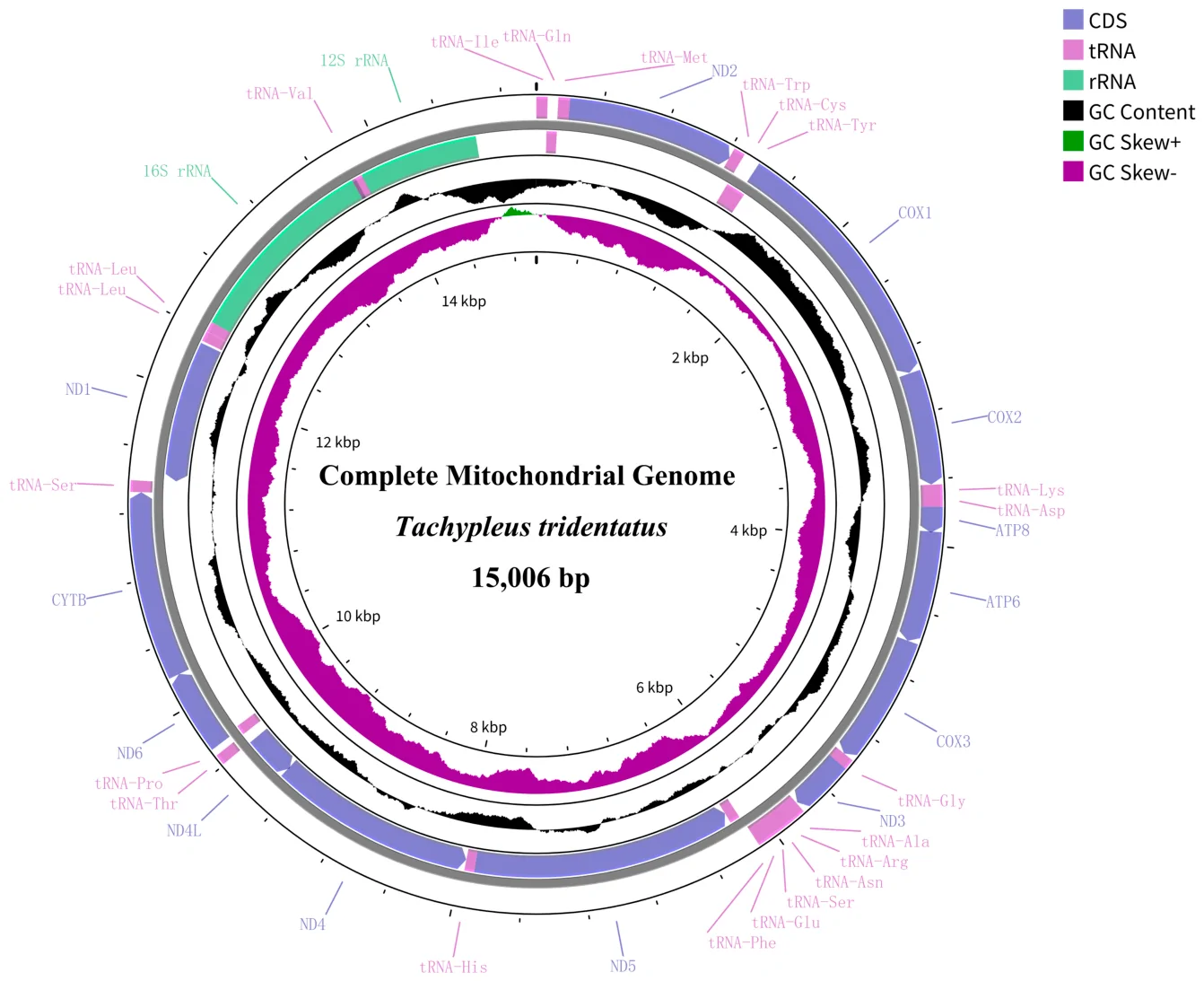
Circular representation of the complete mitochondrial genome of *Tachypleus tridentatus*. The outermost circle represents the genome coordinates, followed by the distribution of protein-coding genes (PCGs), transfer RNA genes (tRNAs), and ribosomal RNA genes (rRNAs) encoded on the heavy (H) and light (L) strands. The inner circles show GC content and GC skew across the mitochondrial genome. The genome is 15,006 bp in length and comprises 13 PCGs, 22 tRNAs, and two rRNAs.

The mitochondrial genome of *T. tridentatus* exhibited the conserved gene content and organization characteristic of horseshoe crab mitochondrial genomes. The circular genome map and detailed gene arrangement are presented in Figure 1. A total of 37 mitochondrial genes were identified, including 13 protein-coding genes (PCGs), 22 transfer RNA genes (tRNAs), and two ribosomal RNA genes (rRNAs) (Figure 1). The 13 PCGs consisted of seven NADH dehydrogenase subunit genes (*ND1*–*ND6* and *ND4L*), three cytochrome c oxidase genes (*COX1*–*COX3*), two ATP synthase genes (*ATP6* and *ATP8*), and one cytochrome b gene (*Cytb*). The mitochondrial genes were encoded on both the heavy (H) and light (L) strands, showing the typical strand distribution pattern observed in metazoan mitochondrial genomes. Most genes were located on the H strand, whereas several genes, including nad6 and a subset of tRNA genes, were encoded on the L strand. The 13 PCGs occupied a large proportion of the mitochondrial genome and exhibited typical mitochondrial genetic characteristics. The lengths of the PCGs ranged from 156 bp (*ATP8*) to 1714 bp (*ND5*). The initiation codons included ATG, ATT, ATC, and TTG. Among the 13 PCGs, *ND5* initiated with TTG, *COX1* with ATC, whereas the remaining genes used ATG or ATT as start codons. Most PCGs terminated with TAA, while *ND4L* terminated with TAG. In addition, three of the 13 PCGs (*COX3*, *ND5*, and *Cytb*; 23.08%) contained incomplete stop codons. These truncated termination codons are expected to be completed at the transcript level through post-transcriptional polyadenylation, generating functional stop codons after the addition of 3′ A residues to the corresponding mRNAs (Table 1).

**Figure 2:**
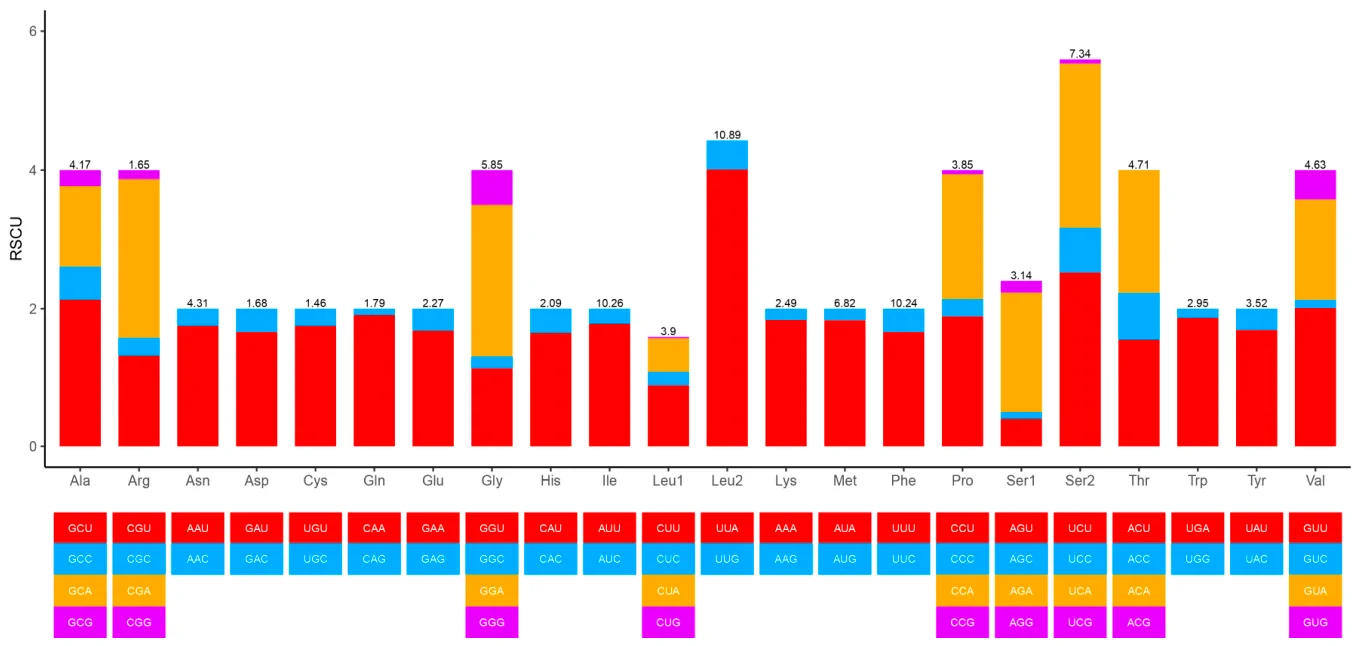
Relative synonymous codon usage (RSCU) analysis of the 13 mitochondrial protein-coding genes (PCGs) of *Tachypleus tridentatus*. The RSCU values of synonymous codons were calculated from the 13 mitochondrial PCGs. Each amino acid is represented by its corresponding synonymous codons, and the bar height indicates the relative usage frequency of each codon. The distribution of RSCU values reflects the synonymous codon usage bias in the mitochondrial genome of *Tachypleus tridentatus*.

To investigate codon usage patterns and amino acid composition, the relative synonymous codon usage (RSCU) and amino acid distribution of the 13 mitochondrial protein-coding genes (PCGs) were analyzed in *T. tridentatus*. The RSCU analysis revealed a biased pattern of synonymous codon usage among the mitochondrial PCGs (Figure 2). Codons corresponding to leucine (Leu), isoleucine (Ile), phenylalanine (Phe), methionine (Met), valine (Val), and glycine (Gly) were preferentially utilized, whereas cysteine (Cys)-encoding codons showed relatively lower usage frequencies. The preferential usage of A/U-ending codons was observed in several frequently used codons, which was consistent with the strong AT bias of the mitochondrial genome. These results indicate that the mitochondrial protein-coding genes of *T. tridentatus* exhibit a distinct codon usage bias.

**Figure 3:**
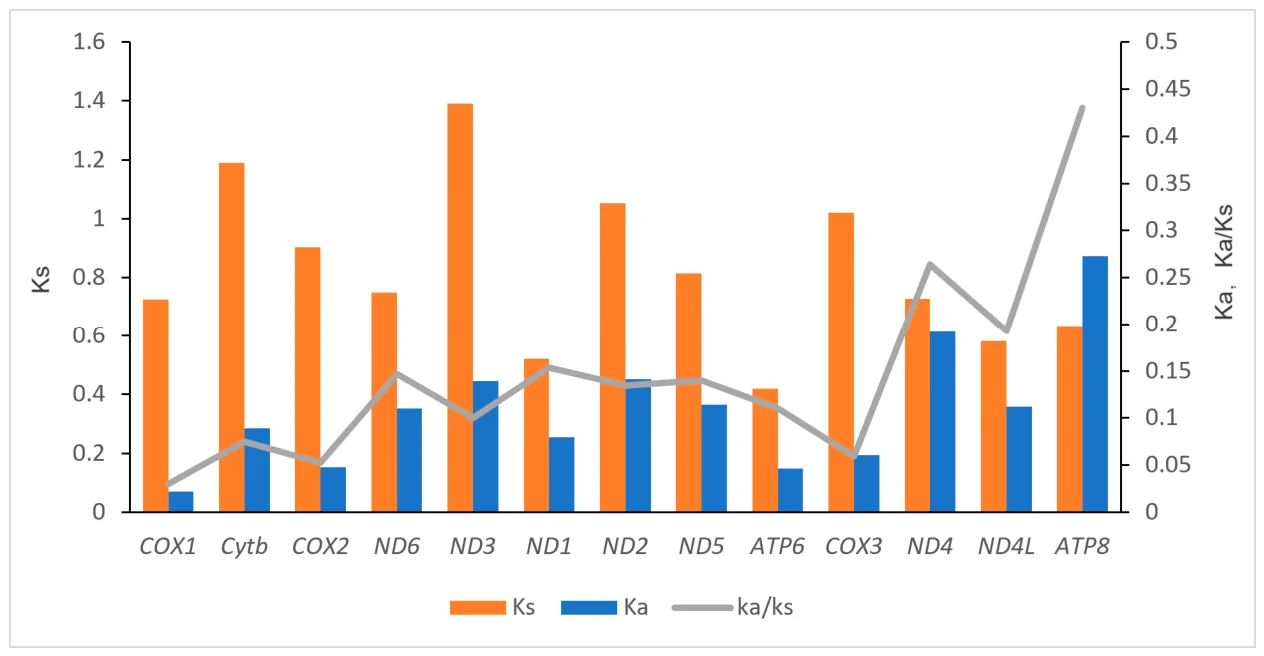
Evolutionary rate analysis of the 13 mitochondrial protein-coding genes of *Tachypleus tridentatus*. The nonsynonymous substitution rate (Ka), synonymous substitution rate (Ks), and Ka/Ks ratio were calculated for each mitochondrial protein-coding gene. Ka/Ks values below 1 indicate that genes have experienced purifying selection during mitochondrial genome evolution.

The selective pressure on the 13 mitochondrial PCGs was evaluated using Ka/Ks ratios (Figure 3). All PCGs showed Ka/Ks values lower than 1, with values ranging from 0.0303 (*COX1*) to 0.4301 (*ATP8*). The lowest Ka/Ks ratio was observed in *COX1*, whereas the highest ratio was detected in *ATP8*. Other genes, including *ND4* and *ND4L*, also showed relatively elevated Ka/Ks values compared with the remaining PCGs. These results indicate that all mitochondrial PCGs of *T. tridentatus* have evolved under purifying selection, although the intensity of selective constraints varies among different genes.

**Figure 4:**
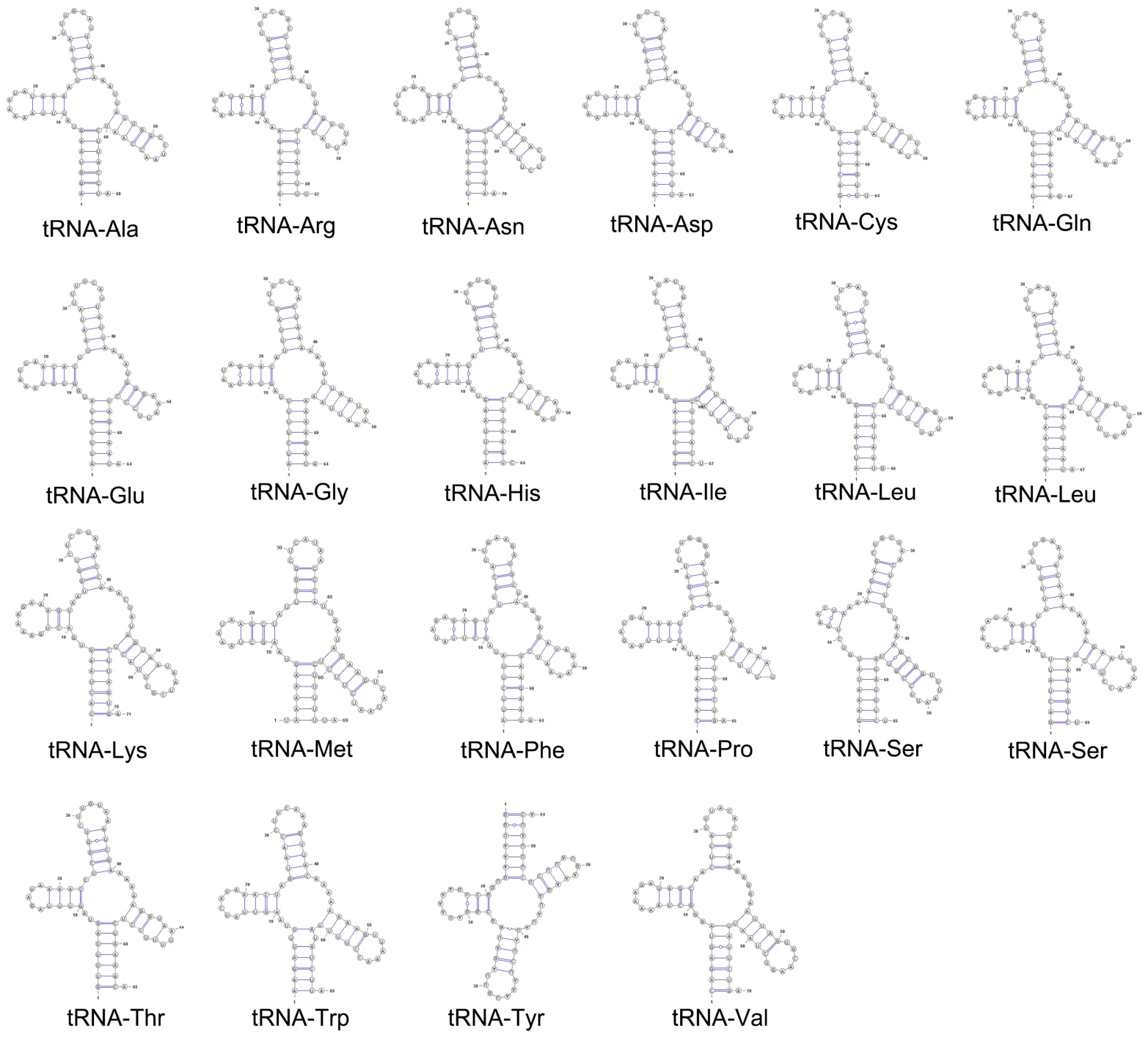
Secondary structure prediction of mitochondrial tRNA genes in *Tachypleus tridentatus*. The predicted secondary structures of the 22 mitochondrial tRNAs are presented. Each tRNA is represented using the conventional cloverleaf model, including the acceptor stem, anticodon arm, and TΨC arm. The identified tRNAs correspond to all 22 mitochondrial tRNA genes encoded by the complete mitochondrial genome of *Tachypleus tridentatus*.

The 22 tRNA genes were distributed throughout the mitochondrial genome and ranged from 62 bp (tRNA-Arg) to 71 bp (tRNA-Lys) in length. The tRNA set contained two leucine tRNAs (tRNA-Leu1 and tRNA-Leu2) and two serine tRNAs (tRNA-Ser1 and tRNA-Ser2), which is consistent with the typical mitochondrial tRNA composition of metazoans. The two ribosomal RNA genes, 16S rRNA and 12S rRNA, were 1326 bp and 779 bp in length, respectively. The 16S rRNA gene was located between tRNA-Leu2 and tRNA-Val, whereas 12S rRNA was located downstream of tRNA-Val. A putative control region was identified between 12S rRNA and the end of the mitochondrial genome, with an estimated length of approximately 391 bp. The secondary structures of the 22 tRNA genes were predicted and exhibited the typical cloverleaf structures of mitochondrial tRNAs (Figure 4). All 22 tRNAs identified in the mitochondrial genome of *T. tridentatus* showed the characteristic organization consisting of the amino acid acceptor arm, anticodon arm, and TΨC arm. The predicted structures included two leucine tRNAs (tRNA-Leu1 and tRNA-Leu2) and two serine tRNAs (tRNA-Ser1 and tRNA-Ser2), which are commonly present in metazoan mitochondrial genomes. The predicted secondary structures of all tRNAs are shown in Figure 4.

### 3.2. Mitochondrial Genetic Divergence Among Extant Genera of Limulidae

**Figure 5:**
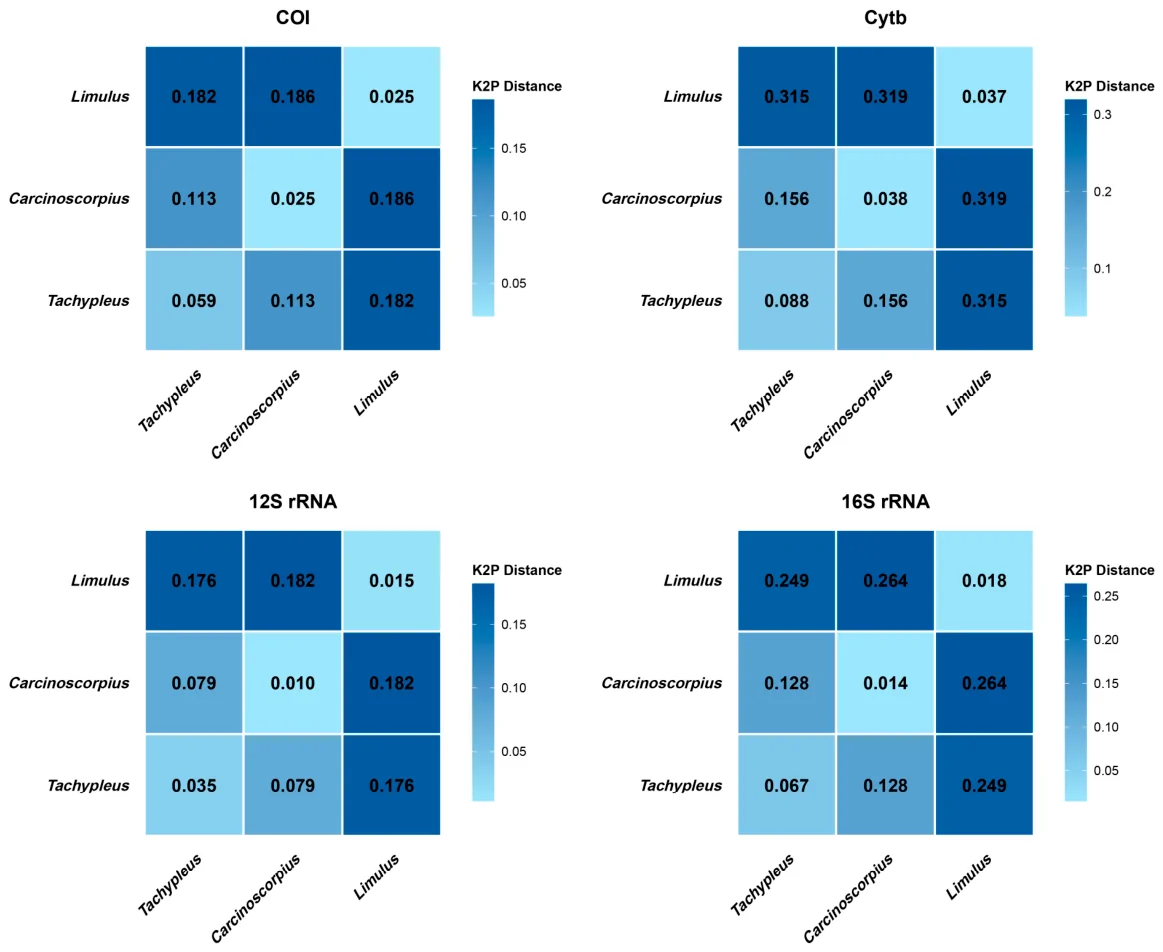
Heatmap of mitochondrial genetic divergence among extant genera of Limulidae based on Kimura two-parameter (K2P) distances. Pairwise genetic distances were calculated among the three extant limulid genera (*Tachypleus*, *Carcinoscorpius*, and *Limulus*) using mitochondrial *COX1*, *Cytb*, 12S rRNA, and 16S rRNA sequences. Color gradients represent the magnitude of K2P genetic distances, with higher values indicating greater mitochondrial sequence divergence. The heatmap illustrates the distinct patterns of intra- and intergeneric genetic differentiation among extant Limulidae genera.

To evaluate mitochondrial genetic divergence among the three extant genera of Limulidae across complementary mitochondrial loci, pairwise genetic distances were calculated using the Kimura two-parameter (K2P) model based on two protein-coding genes (*COX1* and *Cytb*) and two ribosomal RNA genes (12S rRNA and 16S rRNA) (Table S2). The comparative dataset consisted of published mitochondrial genomes representing the extant limulid genera *Tachypleus*, *Carcinoscorpius*, and *Limulus* (Table S1). The K2P distance analyses revealed clear differences in genetic divergence among the three genera, with intergeneric distances consistently higher than intrageneric distances across all examined mitochondrial regions. For the mitochondrial *COX1* gene, the mean genetic distance within *Tachypleus*, *Carcinoscorpius*, and Limulus was 0.059 ± 0.005, 0.025 ± 0.003, and 0.025 ± 0.004, respectively. The intergeneric divergence between *Tachypleus* and *Carcinoscorpius* was 0.113 ± 0.007, whereas the distances between Limulus and the two Asian limulid genera were higher, reaching 0.182 ± 0.009 (*Limulus*–*Tachypleus*) and 0.186 ± 0.011 (*Limulus*–*Carcinoscorpius*) (Table S2; Figure 5). The mitochondrial *Cytb* gene exhibited higher levels of genetic divergence than *COX1*. The mean K2P distances between *Tachypleus* and *Carcinoscorpius*, *Tachypleus* and *Limulus*, and *Carcinoscorpius* and *Limulus* were 0.156 ± 0.010, 0.315 ± 0.018, and 0.319 ± 0.019, respectively. In contrast, the within-genus genetic distances remained relatively low, with values of 0.088 ± 0.006 for *Tachypleus*, 0.038 ± 0.004 for *Carcinoscorpius*, and 0.037 ± 0.006 for *Limulus* (Table S2). The ribosomal RNA genes showed relatively lower genetic divergence compared with protein-coding genes but maintained the same differentiation pattern among genera. For the 12S rRNA gene, the genetic distances between *Tachypleus* and *Carcinoscorpius*, *Tachypleus* and *Limulus*, and *Carcinoscorpius* and *Limulus* were 0.079 ± 0.009, 0.176 ± 0.015, and 0.182 ± 0.016, respectively. Similarly, for the 16S rRNA gene, the corresponding intergeneric distances were 0.128 ± 0.009, 0.249 ± 0.015, and 0.264 ± 0.016, respectively (Table S2). Overall, the K2P distance heatmap showed consistent patterns of mitochondrial divergence among the three extant limulid genera (Figure 5). The genetic distances between Limulus and the two Asian genera (*Tachypleus* and *Carcinoscorpius*) were substantially higher than those between *Tachypleus* and *Carcinoscorpius* across all mitochondrial markers examined. These results indicate distinct mitochondrial differentiation among extant Limulidae genera, with *Tachypleus* and *Carcinoscorpius* exhibiting relatively closer mitochondrial genetic affinity compared with their divergence from *Limulus*.

### 3.3. Mitochondrial Phylogenetic Relationships of Extant Limulidae

**Figure 6:**
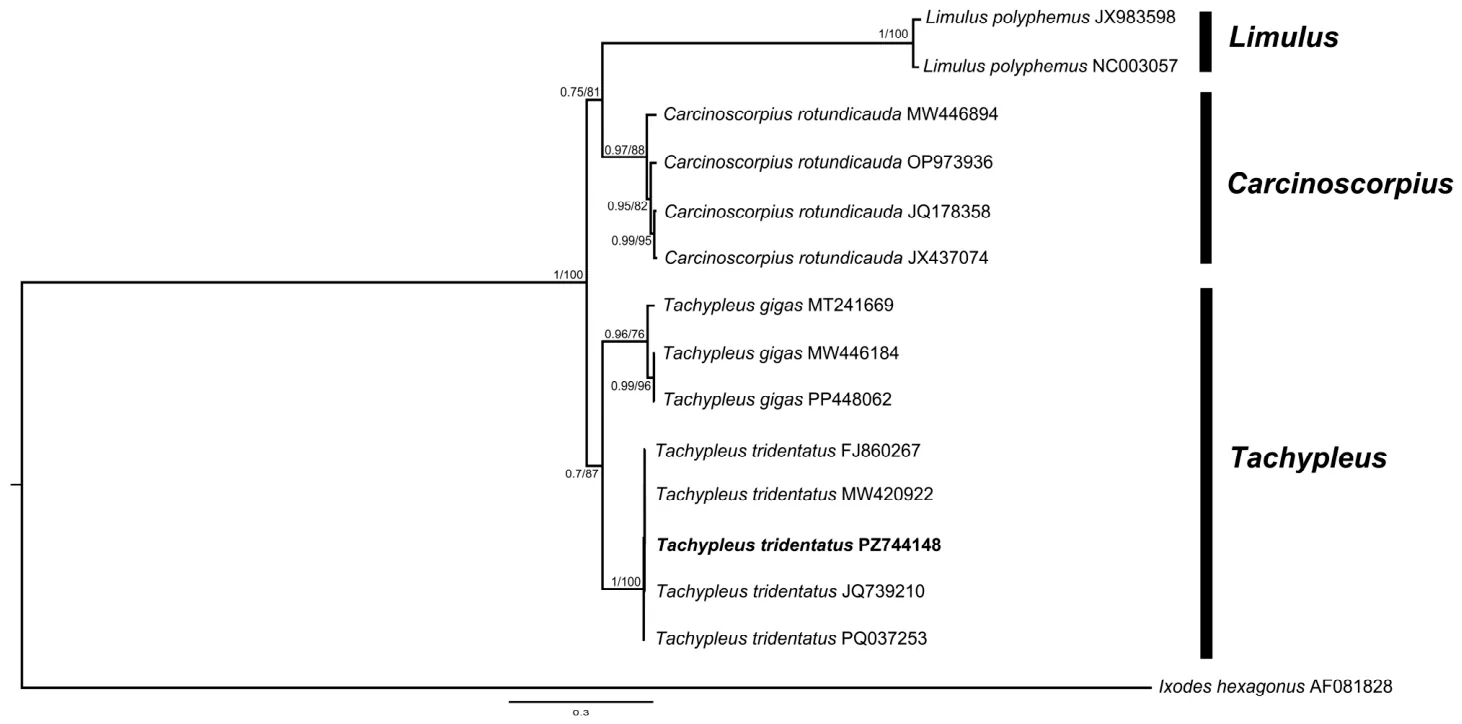
Mitochondrial phylogenetic relationships of extant Limulidae inferred from complete mitochondrial genome sequences using Bayesian inference (BI) and maximum likelihood (ML) methods. The phylogenetic tree was reconstructed based on complete mitochondrial genome sequences from extant limulid taxa, with *Ixodes hexagonus* selected as the outgroup. The topology shown represents the consensus relationship obtained from BI and ML analyses. Numbers at nodes indicate Bayesian posterior probability values and maximum likelihood bootstrap support values, respectively. Support values below 0.7 and for short branches are not shown. The newly sequenced mitochondrial genome of *Tachypleus tridentatus* (PZ744148) is highlighted.

The phylogenetic relationships of extant Limulidae were reconstructed using Bayesian inference (BI) and maximum likelihood (ML) methods based on complete mitochondrial genome sequences. The BI and ML analyses generated highly congruent topologies, consistently recovering three major mitochondrial clades corresponding to the three extant genera, *Carcinoscorpius*, *Tachypleus*, and *Limulus* (Figure 6). The genus *Carcinoscorpius* formed a distinct monophyletic clade in both BI and ML trees. All examined *C. rotundicauda* mitochondrial genomes clustered together, indicating a relatively conserved mitochondrial lineage within this genus. Similarly, *L. polyphemus* sequences formed a separate and well-supported clade, clearly separated from the Asian horseshoe crab genera *Tachypleus* and *Carcinoscorpius*. The genus *Tachypleus* was recovered as a monophyletic clade containing the two sampled species, *T. gigas* and *T. tridentatus*. Within the *Tachypleus* lineage, all *T. tridentatus* mitochondrial genomes, including the newly sequenced mitogenome (*T. tridentatus* PZ744148), clustered together with previously reported *T. tridentatus* sequences. The newly obtained mitochondrial genome showed the closest relationship with *T. tridentatus* MW420922, further supporting its species identification. Both BI and ML analyses recovered a closer relationship between *Tachypleus* and *Carcinoscorpius* than between either Asian genus and *Limulus*. This topology was consistent with the mitochondrial genetic divergence patterns observed in the K2P distance analysis, in which genetic distances between *Tachypleus* and *Carcinoscorpius* were consistently lower than those between these genera and *Limulus*. Overall, the phylogenetic analyses based on two independent approaches (BI and ML) provided consistent evidence for the mitochondrial phylogenetic relationships among extant Limulidae. The results supported the distinct mitochondrial clades corresponding to the three extant genera and confirmed the placement of the newly sequenced *T. tridentatus* mitochondrial genome within the mitochondrial lineage corresponding to *Tachypleus*.

## 4. Discussion

The newly sequenced *T. tridentatus* mitogenome from the Beibu Gulf is highly conserved in overall architecture relative to previously published conspecific mitogenomes. Its length of 15,006 bp, canonical complement of 13 protein-coding genes, 22 tRNAs, and two rRNAs, together with its strong AT bias (74.03%), are consistent with the previously reported mitochondrial characteristics of *T. tridentatus*. These similarities indicate that the conserved genome organization and nucleotide composition observed in the present study are not species- or population-specific innovations. Similarly, the preference for A/U-ending synonymous codons and the consistently low Ka/Ks ratios across the mitochondrial protein-coding genes are compatible with established patterns of mitochondrial codon usage and purifying selection. These findings therefore primarily confirm the conserved structural and evolutionary characteristics of the mitochondrial genome in *T. tridentatus*, rather than representing newly discovered genome-level features [36,56,57]. From an ecological perspective, the conserved mitochondrial architecture may be relevant to the environmental conditions experienced by *T. tridentatus*. As a benthic and intertidal species, *T. tridentatus* inhabits coastal environments characterized by fluctuations in temperature, salinity, oxygen availability, and other physicochemical conditions. Because mitochondrial oxidative phosphorylation is central to aerobic energy production, conservation of mitochondrial genes encoding respiratory-chain components may be functionally relevant under such variable conditions [22,58]. The mitochondrial genome of *T. tridentatus* also showed a pronounced AT bias, accompanied by a slightly positive AT-skew and a negative GC-skew. These values are broadly consistent with the AT-rich composition reported for horseshoe crab mitochondrial genomes and therefore do not represent a lineage-specific feature unique to the present specimen. Nevertheless, the high AT content provides a useful compositional characteristic for comparative analyses within Limulidae and is consistent with the A/U-ending codon preference observed across the 13 PCGs [59,60,61,62]. In *T. tridentatus*, this conservation is particularly relevant because all 13 mitochondrial PCGs contribute to essential components of oxidative phosphorylation, including NADH dehydrogenase, cytochrome c oxidase, cytochrome b, and ATP synthase complexes. The functional importance of these genes is consistent with the sequence constraints observed in the present dataset. However, the observed sequence conservation alone cannot establish a direct link to adaptation to the intertidal environment [23,63]. The predicted secondary structures of all 22 tRNAs further support this conservation. All tRNAs retained the characteristic mitochondrial tRNA organization, including duplicated serine (tRNA-Ser1 and tRNA-Ser2) and leucine (tRNA-Leu1 and tRNA-Leu2) tRNAs typical of metazoan mitochondrial genomes [64,65].

The RSCU analysis further revealed a strong preference for A/U-ending synonymous codons, which is consistent with the high AT content of the *T. tridentatus* mitogenome. The preferential use of amino acids such as leucine, isoleucine, phenylalanine, methionine, valine, and glycine is broadly comparable with patterns reported for other arthropod mitochondrial genomes and should therefore not be regarded as a species-specific signature. More informative for *T. tridentatus* is the variation in Ka/Ks ratios among individual PCGs. All 13 PCGs had Ka/Ks ratios below one, which is consistent with predominant purifying selection, although the magnitude of sequence constraint varied among genes. *COX1* showed the lowest Ka/Ks ratio, whereas *ATP8*, *ND4*, and *ND4L* exhibited relatively higher values. This variation indicates heterogeneity in evolutionary rates among mitochondrial PCGs. The particularly low Ka/Ks ratio of *COX1* is consistent with its essential role in cytochrome c oxidase and helps explain its long-standing utility as a molecular marker for species identification and evolutionary studies [23,66,67,68,69]. Thus, the main significance of the present Ka/Ks results is not simply confirmation that mitochondrial genes are generally conserved, but the documentation of gene-specific differences in selective constraint within the *T. tridentatus* mitogenome.

Comparative analyses of mitochondrial genomes provide valuable insights into lineage divergence and mitochondrial phylogenetic relationships among extant horseshoe crabs. In the present study, genetic divergence was evaluated using four complementary mitochondrial markers, including two protein-coding genes (*COX1* and *Cytb*) and two ribosomal RNA genes (12S rRNA and 16S rRNA). This marker combination was selected to capture variation across mitochondrial regions with different levels of sequence conservation. It also reduced reliance on a single mitochondrial locus when evaluating intergeneric divergence. The results showed that *Cytb* exhibited greater genetic divergence than *COX1*, whereas the two rRNA genes generally showed lower levels of divergence than the protein-coding genes. Importantly, despite these differences in the magnitude of genetic divergence, all four markers consistently recovered the same overall pattern of differentiation among the three extant limulid genera. The concordance between protein-coding and rRNA markers therefore provides stronger support for the observed intergeneric differentiation than would reliance on a single mitochondrial marker. Across all four markers, intergeneric divergence was substantially greater than intrageneric variation, indicating pronounced mitochondrial genetic differentiation among the three extant genera. The congruent patterns observed in both protein-coding and ribosomal RNA genes further demonstrate that complete mitochondrial genomes contain robust phylogenetic signals for resolving mitochondrial phylogenetic relationships within Limulidae. The K2P genetic distance analyses consistently showed that *Tachypleus* and *Carcinoscorpius* were genetically more similar to each other than either was to *Limulus*. This pattern was particularly evident in the protein-coding genes, where the American horseshoe crab (*Limulus polyphemus*) exhibited markedly greater divergence from the two Asian genera. Similar phylogenetic relationships have been reported in previous molecular studies based on mitochondrial and nuclear markers, which consistently placed Limulus as a distinct Atlantic lineage and *Tachypleus* plus *Carcinoscorpius* within the Indo-West Pacific lineage [34,35,70,71]. These results support pronounced differentiation among mitochondrial lineages within Limulidae. Nevertheless, the magnitude of K2P distance should not be interpreted as direct evidence of reproductive isolation, demographic independence, or species boundaries, because sequence divergence at mitochondrial loci represents only one component of species-level differentiation. Differences in nuclear genomes, morphology, ecology, and population connectivity may provide additional or contrasting evidence. Therefore, the present results are interpreted conservatively as evidence of mitochondrial genetic divergence among the extant limulid genera.

The phylogenetic relationships inferred from Bayesian inference (BI) and maximum likelihood (ML) analyses were highly congruent and further supported the genetic divergence patterns revealed by the K2P analyses. Both phylogenetic methods recovered three well-supported monophyletic lineages corresponding to *Limulus*, *Tachypleus*, and *Carcinoscorpius*, with strong nodal support throughout the tree topology. The newly sequenced *T. tridentatus* mitogenome from the Beibu Gulf clustered robustly with previously published *T. tridentatus* mitochondrial genomes, supporting its taxonomic identity and the reliability of the genome assembly and annotation. However, this phylogenetic clustering should not be interpreted as evidence that the Beibu Gulf population is genetically indistinguishable from other geographic populations. Previous population-level mitochondrial DNA studies have examined *T. tridentatus* from multiple localities along the Chinese coast, including Guangxi, Fujian, Zhejiang, and Hainan, as well as populations from the Taiwan Strait and Japan, and have generally reported relatively low mitochondrial differentiation across much of the species’ distribution, although significant differentiation has been detected between some geographically separated populations [39,40]. Therefore, the available evidence does not currently support the conclusion that the Beibu Gulf population represents a uniquely divergent mitochondrial lineage. Instead, the complete mitogenome generated in the present study provides an important regional reference for the Beibu Gulf and expands the geographic coverage of mitochondrial genomic resources for *T. tridentatus*. Because previous geographic studies have mainly relied on partial mitochondrial markers, additional complete mitogenomes from geographically separated populations will be necessary to determine whether region-specific mitochondrial variants or haplotypes occur within the species. Compared with previous studies that primarily described individual mitochondrial genomes or reconstructed phylogenetic relationships using limited mitochondrial markers, the present study integrates complete mitochondrial genome sequencing with comparative analyses of four mitochondrial markers across all extant limulid genera. The consistency between K2P genetic distances and BI/ML phylogenetic topologies demonstrates the utility of complete mitogenomes for resolving mitochondrial phylogenetic relationships among horseshoe crabs and provides a comparative framework for assessing mitochondrial divergence within Limulidae. The newly generated mitochondrial genome was obtained from an F1 *T. tridentatus* individual produced from wild-caught broodstock maintained at an aquaculture facility in Guangxi, a region adjacent to the Beibu Gulf. As the F1 offspring were derived directly from wild-caught parents, they are less likely to have experienced the cumulative genetic effects associated with prolonged captive propagation, such as genetic drift and artificial selection over multiple generations. Nevertheless, the mitochondrial haplotype represented by a single F1 individual reflects its maternal lineage and may not capture the full mitochondrial diversity of wild *T. tridentatus* populations in the Beibu Gulf region. Therefore, the mitogenome generated in this study should be regarded as a regional reference genomic resource rather than a population-level genetic baseline. The clustering of the newly sequenced mitogenome with previously reported *T. tridentatus* mitogenomes from other geographic regions indicates a conserved mitochondrial lineage among the available conspecific sequences. However, because mitochondrial genomes are maternally inherited, this pattern should be interpreted as evidence of shared maternal ancestry rather than direct evidence of contemporary population connectivity. Broader sampling of wild individuals and multiple maternal lineages, together with nuclear genomic markers such as single nucleotide polymorphisms (SNPs) or microsatellites, will be necessary to resolve fine-scale geographic population structure, demographic history, and gene flow throughout the distribution range of *T. tridentatus*.

The phylogenetic reconstruction further revealed a clear mitochondrial divergence between the Atlantic *L. polyphemus* lineage and the Indo-West Pacific *Tachypleus* and *Carcinoscorpius* lineages, consistent with the long-term geographic separation of these major horseshoe crab groups. However, this pattern should be interpreted specifically as a mitochondrial phylogenetic signal rather than definitive evidence of genome-wide species relationships. Mitochondrial genomes are predominantly maternally inherited and represent a single, non-recombining maternal lineage; consequently, mitochondrial genealogies may differ from nuclear species histories. Such mitochondrial–nuclear discordance can arise through sex-biased dispersal, incomplete lineage sorting, historical demographic processes, or mitochondrial introgression. In particular, introgression or retention of ancestral mitochondrial haplotypes could cause mitochondrial lineages to appear more closely related or more deeply divergent than the corresponding nuclear histories. Therefore, the Atlantic–Indo-West Pacific pattern recovered here should primarily be interpreted as evidence for differentiation of mitochondrial maternal lineages and historical biogeographic structure, rather than as definitive evidence for genome-wide population or species divergence. The concordance of the mitochondrial phylogeny with the known geographic distribution of extant horseshoe crab groups provides a useful historical framework for mitochondrial biogeographic inference [14,35,72]. However, multilocus nuclear markers and genome-wide SNP data will be required to test whether these mitochondrial relationships accurately represent the broader species-level evolutionary history and to clarify the timing and mechanisms of diversification within Limulidae.

*T. tridentatus* has experienced substantial population declines throughout much of its distribution owing to habitat loss, coastal reclamation, pollution, and overexploitation. Because successful reproduction depends on intact intertidal spawning beaches and adjacent nursery habitats, continued degradation of coastal ecosystems poses a major threat to the long-term persistence of natural populations [18,41]. Consequently, conservation strategies should prioritize the protection and restoration of critical coastal habitats while incorporating molecular genetic information into management programmes. The complete mitochondrial genome generated in this study provides a regional molecular reference for species identification, comparative mitogenomics, and future conservation-genetic studies of *T. tridentatus*. The pronounced mitochondrial differentiation observed among the three extant limulid genera highlights their distinct mitochondrial lineage patterns and provides comparative context for future conservation-genetic studies. For *T. tridentatus*, previous population-level mitochondrial studies have generally indicated relatively weak differentiation across much of its geographic range, although localized genetic differences have been detected among some geographically separated populations. Recent genomic research has further demonstrated that geographic and climatic dynamics can shape population genetic structure and conservation vulnerability in Asian horseshoe crabs, emphasizing the importance of considering both genetic connectivity and environmental heterogeneity in regional conservation planning [20]. Therefore, future conservation planning should prioritize the protection of geographically representative populations and their associated spawning and nursery habitats rather than relying on a single regional population as a proxy for the species as a whole. In particular, the Beibu Gulf region should be incorporated into future regional genetic monitoring programs, with the complete mitogenome generated in this study serving as a reference for future surveys of mitochondrial haplotype variation. Where geographically restricted haplotypes or genetically differentiated populations are identified, these populations should be prioritized for in situ conservation. Indiscriminate translocation or mixing of individuals among geographically separated populations should be avoided until their genetic compatibility has been evaluated. Maintaining connectivity among suitable coastal habitats is also important for preserving natural gene flow and population persistence [73]. Recent population genetic work on *T. tridentatus* has demonstrated the practical value of genetic differentiation for management-unit delineation, with geographically differentiated populations in Palawan being proposed as two management units [74]. However, formal designation of management units (MUs) or evolutionarily significant units (ESUs) should not be based on mitochondrial data alone. Because mitochondrial genomes represent a maternally inherited genetic history, nuclear markers or genome-wide SNP data should be integrated with mitochondrial variation to confirm population boundaries and evaluate genome-wide differentiation. Thus, the present mitogenome provides a regional genetic baseline for the Beibu Gulf and a foundation for future population monitoring, whereas broader genomic sampling will be necessary to translate geographic genetic differentiation into formally defined conservation and management units.

## 5. Conclusions

This study provides a complete mitochondrial genome of an F1 *T. tridentatus* individual derived from wild-caught broodstock from the Beibu Gulf and expands the geographic representation of available mitogenomic resources for this species. Comparison with previously published *T. tridentatus* mitogenomes indicates that the major structural and compositional characteristics of the Beibu Gulf-derived mitogenome are highly conserved, confirming rather than substantially revising the established mitochondrial features of the species. The principal contribution of this study is the integration of the new regional mitogenome with comparative complete mitochondrial genomes and four mitochondrial markers representing all extant limulid genera. The concordance of K2P genetic-distance patterns with BI and ML phylogenies provides consistent evidence for mitochondrial genetic differentiation among the three extant limulid genera and supports the closer mitochondrial relationship between *Tachypleus* and *Carcinoscorpius* than between either genus and *Limulus*. Because these inferences are based exclusively on maternally inherited mitochondrial genomes, they should not be interpreted as definitive evidence of species–tree relationships or genome-wide lineage diversification. The present mitochondrial dataset alone also cannot resolve population structure, contemporary gene flow, or demographic history within *T. tridentatus*. Integration of independently inherited nuclear markers and genome-wide SNP data will therefore be necessary to evaluate mitochondrial–nuclear concordance and to resolve these population-level evolutionary processes. Accordingly, the newly generated mitogenome should primarily be regarded as a regional mitochondrial reference resource for species identification, comparative mitogenomics, and future population- and conservation-genetic studies of *T. tridentatus*, rather than as evidence sufficient to resolve population connectivity, demographic history, or genome-wide evolutionary relationships.

## Figures and Tables

**Table 1:** Characteristic constituents of the mitochondrial genome of *Tachypleus tridentatus*.

Feature	Strand	Position	Length	Initiation/Stop Codon
tRNA-Ile	H	1–67	67	
tRNA-Gln	L	132–66	67	
tRNA-Met	H	131–199	69	
NADH dehydrogenase subunit 2	H	200–1219	1020	ATT/TAA
tRNA-Trp	H	1218–1286	69	
tRNA-Cys	L	1351–1289	63	
tRNA-Tyr	L	1415–1352	64	
cytochrome c oxidase subunit 1	H	1387–2946	1560	ATC/TAA
cytochrome c oxidase subunit 2	H	2950–3633	684	ATG/TAA
tRNA-Lys	H	3635–3705	71	
tRNA-Asp	H	3705–3767	63	
ATP synthase F0 subunit 8	H	3768–3923	156	ATT/TAA
ATP synthase F0 subunit 6	H	3917–4591	675	ATG/TAA
cytochrome c oxidase subunit 3	H	4591–5374	784	ATG/TAA
tRNA-Gly	H	5375–5438	64	
NADH dehydrogenase subunit 3	H	5439–5783	345	ATT/TAA
tRNA-Ala	H	5790–5857	68	
tRNA-Arg	H	5858–5919	62	
tRNA-Asn	H	5919–5988	70	
tRNA-Ser	H	5989–6053	65	
tRNA-Glu	H	6054–6117	64	
tRNA-Phe	L	6192–6126	67	
NADH dehydrogenase subunit 5	L	7906–6193	1714	TTG/TAA
tRNA-His	L	7970–7907	64	
NADH dehydrogenase subunit 4	L	9309–7972	1338	ATG/TAA
NADH dehydrogenase subunit 4L	L	9602–9303	300	ATG/TAG
tRNA-Thr	H	9605–9669	65	
tRNA-Pro	L	9735–9671	65	
NADH dehydrogenase subunit 6	H	9712–10,200	489	ATT/TAA
cytochrome b	H	10,200–11,328	1129	ATG/TAA
tRNA-Ser	H	11,329–11,397	69	
NADH dehydrogenase subunit 1	L	12,324–11,407	918	ATG/TAA
tRNA-Leu	L	12,405–12,340	66	
tRNA-Leu	L	12,473–12,407	67	
16S ribosomal RNA	L	13,792–12,467	1326	
tRNA-Val	L	13,837–13,768	70	
12S ribosomal RNA	L	14,615–13,837	779	

H and L refer to the heavy and light strand, respectively.

## Data Availability

The genome sequence data that support the findings of this study are openly available in GenBank of NCBI at [https://www.ncbi.nlm.nih.gov] under the accession no. PZ744148. The associated BioProject, Bio-Sample, and SRA numbers are PRJNA1496527, SAMN61723888, and SRR39699097, respectively.

## Supplementary Materials

The following supporting information can be downloaded at: https://www.mdpi.com/article/10.3390/ani16182823/s1, Table S1. The details of deposited mitochondrial genomes in Genbank used in this research. Table S2. Intra- and intergeneric genetic divergence among the three extant genera of Limulidae (*Tachypleus*, *Carcinoscorpius*, and *Limulus*) estimated using Kimura two-parameter (K2P) distances based on mitochondrial genes *COI*, *Cytb*, 12S rRNA, and 16S rRNA. Figure S1. Distribution map of sequencing depth of the mitochondrial genome of *Tachypleus tridentatus*.
