# Supplementary material for: Characterization of the Complete Mitochondrial Genome of Tachypleus tridentatus and Mitochondrial Phylogenetic Analysis of Extant Limulidae
Source: Animals (Basel). 2026 Sep 8;16(18):2823. doi: 10.3390/ani16182823 (PMC13603792; doi:10.3390/ani16182823)
Supplement: Supplementary file 1 [file animals-16-02823-s001.zip › Table S1.pdf]

Table S1. The details of deposited mitochondrial genomes in Genbank used in this research.

**Accession numbers**	**Morphological type**	**Collection location**	**References or collector**	**Date**
MW446184	*Tachypleus gigas*	Malaysia: Kemaman, Terengganu	Sarmiento et al.	2021
PP448062	*Tachypleus gigas*	Malaysia: Kota Belud, Sabah	Kasmat et al.	2024
MT241669	*Tachypleus gigas*	the East Coast Park, Singapore	Shingate et al.	2020
JQ178358	*Carcinoscorpius rotundicauda*	South Korea	Baek et al.	2014
JX437074	*Carcinoscorpius rotundicauda*	Fujian, China	Weng et al.	2013
OP973936	*Carcinoscorpius rotundicauda*	Malaysia: Sabah	Kumar et al.	2023
MW446894	*Carcinoscorpius rotundicauda*	Indonesia: North Jakarta	McDonald et al.	2021
JQ739210	*Tachypleus tridentatus*	South Korea	Baek et al.	2014
MW420922	*Tachypleus tridentatus*	East China Sea	Pan et al.	2023
PQ037253	*Tachypleus tridentatus*	Malaysia: Sandakan, Sabah	Kumar et al.	2024
FJ860267	*Tachypleus tridentatus*	Fujian, China	Weng et al.	2012
JX983598	*Limulus polyphemus*	USA: Jackson Estuarine Lab, Durham, NH	Chabot and Cooper	2012
AF216203	*Limulus polyphemus*	USA: Gulf Coast of Florida	Lavrov et al.	2016
