# Supplementary material for: Characterization of the Complete Mitochondrial Genome of Tachypleus tridentatus and Mitochondrial Phylogenetic Analysis of Extant Limulidae
Source: Animals (Basel). 2026 Sep 8;16(18):2823. doi: 10.3390/ani16182823 (PMC13603792; doi:10.3390/ani16182823)
Supplement: Supplementary file 1 [file animals-16-02823-s001.zip › Table S2.pdf]

Table S2 Intra- and intergeneric genetic divergence among the three extant genera of Limulidae (*Tachypleus*, *Carcinoscorpius*, and *Limulus*) estimated using Kimura two-parameter (K2P) distances based on mitochondrial genes COI, Cytb, 12S rRNA, and 16S rRNA.

cytochrome c oxidase subunit I			
	Tachypleus	*Carcinoscorpius*	*Limulus*
Tachypleus	0.059±0.005		
Carcinoscorpius	0.113±0.007	0.025±0.003	
Limulus	0.182±0.009	0.186±0.011	0.025±0.004
cytochrome b			
	Tachypleus	*Carcinoscorpius*	*Limulus*
Tachypleus	0.088±0.006		
Carcinoscorpius	0.156±0.01	0.038±0.004	
Limulus	0.315±0.018	0.319±0.019	0.037±0.006
12S rRNA			
	Tachypleus	*Carcinoscorpius*	*Limulus*
Tachypleus	0.035±0.005		
Carcinoscorpius	0.079±0.009	0.01±0.002	
Limulus	0.176±0.015	0.182±0.016	0.015±0.004
16S rRNA			
	Tachypleus	*Carcinoscorpius*	*Limulus*
Tachypleus	0.067±0.005		
Carcinoscorpius	0.128±0.009	0.014±0.002	
Limulus	0.249±0.015	0.264±0.016	0.018±0.004
